# Ileal perforation peritonitis secondary to ingestion of magnetic beads in the older child: A case report

**DOI:** 10.1016/j.ijscr.2024.109915

**Published:** 2024-06-19

**Authors:** Ndèye Aby Ndoye, Ibrahima Bocar Welle, Bembo Lamega, Amadou Diawara, Florent Tshibwid A. Zeng, Gabriel Ngom

**Affiliations:** a Department of Pediatric Surgery, Albert Royer National Children's Hospital Center, Université Cheikh Anta Diop, Dakar, Senegal; b Department of Pediatric Surgery, Albert Royer National Children's Hospital Center, Dakar, Senegal

**Keywords:** Intestinal perforation, Foreign body, Magnets, Child, Case report

## Abstract

**Introduction and importance:**

Foreign body ingestion is frequent in younger children, with generally good outcome on conservative management. However, magnetic beads ingestion is an exceptional cause of intestinal perforation in the older children.

**Case presentation:**

An 8-year-old boy presented with clinical signs of generalized acute peritonitis. Abdominal plain X-ray confirmed the foreign object in the digestive tract and oriented the etiology by highlighting several air-fluid levels, distended small bowel loops, pneumoperitoneum and the presence of a bilobed foreign body projected adjacent to the 5th lumbar vertebra. Open surgical exploration was performed and revealed a peritoneal fluid, 2 perforations in the small bowel and 2 adhered pieces of magnets. A 20 cm ileal resection, including the segment with the 2 perforations, was performed followed by a terminal ileostomy. The restoration of gastrointestinal continuity was performed 16 days later. After a follow-up of 2 years and 8 months, the patient was free of any symptom.

**Clinical discussion:**

In cases of acute peritonitis due to perforation, the general condition deteriorates progressively. Fever may be absent, as was the case with our patient. Abdominal pain is the predominant symptom, it is often accompanied by vomiting that can be alimentary, bilious, or even fecaloid and/or by cessation of bowel movements and/or gas. Abdominal rigidity is a major physical sign, sometimes replaced by generalized guarding.

**Conclusion:**

Ingestion of gastrointestinal foreign bodies is rare in older children, the presence of more than one magnet can lead to peritonitis due to intestinal perforation.

## Highlights

• What is known on the subject? Intestinal perforation due to magnets ingestion is not exceptional in children. It happens when more than one magnet is ingested. However, it is not the most common etiology of acute abdomen in children.
• What this study adds? Peritonitis due to intestinal perforation secondary to magnets ingestion may be confirmed only during surgical exploration in some cases. In some cases, the management may require multiple surgeries.
• Open surgery is still used in developing countries

## 1 Introduction

The ingestion of foreign bodies (FBs) is a common reason for pediatric outpatient consultation or emergency admissions. In most cases, it is benign, without consequences and occurs in the context of domestic accidents, in the young children [1,2]. Indeed, the majority of these FBs are eliminated through intestinal tract, and less than 1 % of them require surgical treatment [3].

Perforation of an intra-digestive hollow organ by a FB can be attributed to caustic substances, sharp objects, of exceptionally, magnetic pieces.

We report a case of peritonitis by ileal perforation, secondary to magnets ingestion in an 8-year-old boy, along with a short- and mid-term outcomes. The SCARE guidelines were followed [4].

## 2 Case description

An 8-year-old boy presented to a healthcare center with severe abdominal pain persisting for a week, associated with post-prandial food vomiting and intermittent fever. There were no reports of diarrhea or bowel obstruction. Symptomatic treatment was prescribed but unsuccessful leading to referral to our department for further management. Medical history noted epilepsy which had been monitored and treated up for 3 years.

On admission, the temperature was 37 °C, and the patient exhibited tachycardia at 126 beats per min. Physical examination revealed a good general condition, with normal-colored non jaundiced mucous membranes, and no signs of dehydration. The abdomen was distended and tender upon palpation, with a generalized contraction and umbilical pain.

Blood test revealed hyperleukocytosis with neutrophils at 27,240 elements/ml, hypochromic microcytic anemia with hemoglobin at 10.8 g/dl. The blood electrolytes revealed hyponatremia (123 mmol/L) and hypochloremia (84 mmol/L). The prothrombin rate was reduced to 55.2 %.

**Fig. 1:**
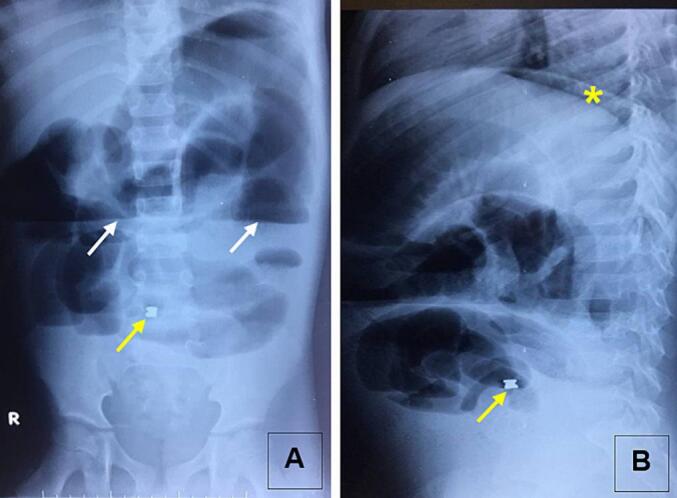
Abdominal plain X-ray on anteroposterior (A) and lateral (B) views. Yellow arrows: foreign bodies, white arrows: air-fluid levels, and yellow asterisk: pneumoperitoneum. (For interpretation of the references to colour in this figure legend, the reader is referred to the web version of this article.)

Abdominal ultrasound did not provide significant findings, except for moderate colonic distension. A standing anteroposterior abdominal X-ray without contrast revealed multiple mixed air-fluid levels, distended intestinal loops, and a bilobed foreign body projecting near the 5th lumbar vertebra (L5). A lateral view of the X-ray confirmed the presence of pneumoperitoneum (Fig. 1).

**Fig. 2:**
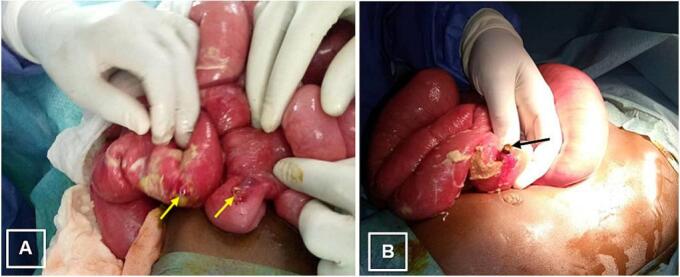
Intraoperative image. This shows two small bowel perforations (A) and two joined magnetic pieces (B).

Based on the suspicion of peritonitis caused by a gastrointestinal foreign body and signs of organ perforation, emergency surgery was indicated. The patient underwent preoperative resuscitation, including rehydration, correction of electrolyte imbalances, transfusion of fresh frozen plasma, and antibiotic prophylaxis. Surgery was performed by a Professor in Pediatric Surgery through a transverse supraumbilical laparotomy was performed. Intraoperative exploration of the abdominal cavity revealed 700 cc of citrine yellow fluid, two perforations in the small intestine at 20 cm and 70 cm from the ileocecal junction, with dilation of the proximal intestinal loops. Two attached magnets were found at the site of the first perforation (Fig. 2).

**Fig. 3:**
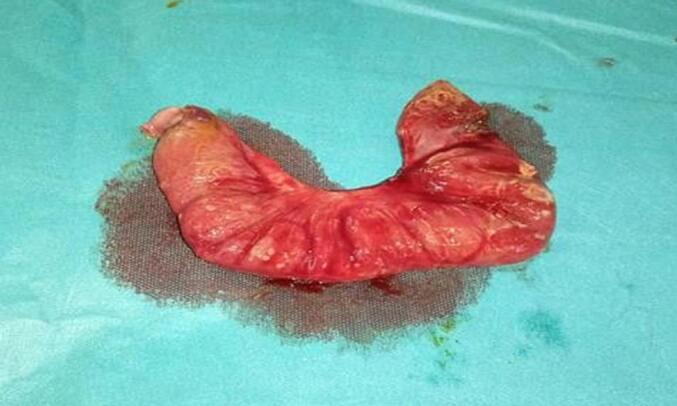
Ileal resection. Resection of 20 cm of ileum, removing both perforations.

**Fig. 4:**
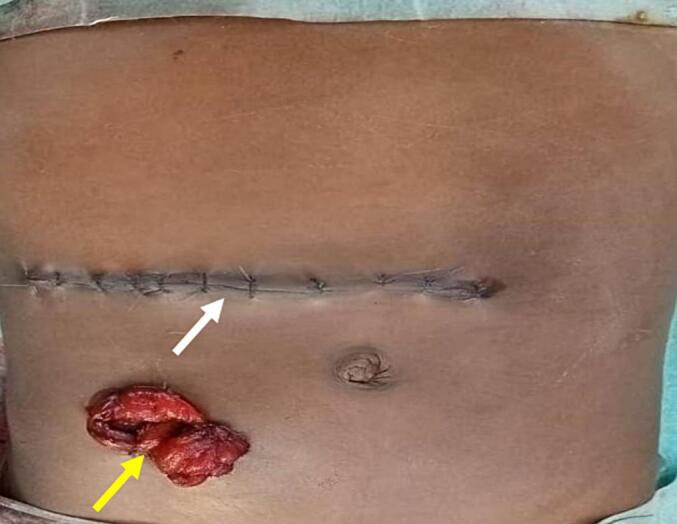
Abdominal closure and ileostomy. Abdominal appearance after abdominal closure of the transverse approach (white arrow) and terminal ileostomy confection (yellow arrow). (For interpretation of the references to colour in this figure legend, the reader is referred to the web version of this article.)

A 20 cm ileal resection (Fig. 3), removing the 2 perforations was performed followed by a terminal loop ileostomy (Fig. 4). The abdominal cavity was thoroughly irrigated with warm isotonic saline before layered closure. The patient's condition improved, with good overall health, a productive stoma observed the day after surgery, and correction of minor hydroelectrolytic imbalances. Restoration of gastrointestinal continuity was performed 16 days after the initial surgery.

Macroscopic examination of the surgical specimen revealed two perforation zones measuring between 1.5 cm and 0.5 cm in length, along with fragments covered with false membranes. Microscopically, there was a suppurative necrotic area of the mucosa showing a moderate leukocytic infiltrate, indicating suppurative ileitis with intense edematous-congestive reaction.

After a hospitalization period of 27 days, the patient was discharged. Follow-up evaluation after 2 years and 8 months showed the patient was asymptomatic, and psychological assessment did not reveal any abnormalities.

## 3 Discussion

The ingestion of digestive foreign bodies is a relatively common reason for pediatric consultations. However, the outcome is often favorable with conservative management and without complications, as the foreign body is eliminated through the intestinal transit. The outcome is generally influenced by the type of ingested foreign body. Most commonly, these include coins, beads, small toys, and sharp objects. Ingestion of magnetic objects is rarer [5,7,8].

Foreign body ingestion primarily occurs in infants and young children, with a peak incidence between 6 months and 6 years of age. This can be attributed to various factors, including the psychomotor development of young children, their natural inclination to explore their surroundings, and the reflex they have to put objects in their mouths. Additionally, several studies have found a significant male predominance [2,7,9]. Our patient is a boy whose age is older than what is typically observed in cases of foreign body ingestion. This prompted us to conduct a psychological evaluation, which revealed no abnormalities.

In cases of acute peritonitis due to perforation, the general condition is initially preserved but deteriorates as the clinical picture progresses. Fever may be absent, as was the case with our patient. Abdominal pain is the predominant symptom, characterized by sudden, intense, and sometimes paroxysmal onset. Its initial location and intensity provide some localization value, but it quickly spreads to involve the entire abdomen. It is often accompanied by gastrointestinal disturbances, including vomiting that can be alimentary, bilious, or even fecaloid. Complete cessation of bowel movements and/or gas occurs when there is complete intestinal paralysis. Physical examination is crucial in guiding therapeutic decisions, especially in resource-limited settings where specific diagnostic tests may not be readily available. Abdominal rigidity is a major physical sign, sometimes replaced by generalized guarding. Other signs include tenderness at the umbilicus and in the Douglas pouch [10]. These signs were observed in our patient.

In suspicion of acute abdomen in children, abdominal ultrasound is the first line imaging investigation. It is a non-invasive examination, but its usefulness may be limited by intestinal distension. In our patient, abdominal ultrasound did not provide significant findings. In some cases, abdominal scanner can provide more information on etiology of perforation. It however irradiant and not always available in emergency in resource-constrained settings. Plain abdominal X-ray is sometimes sufficient for diagnosis and can help identify the etiology. In our case, the plain X-ray revealed the digestive foreign body in addition to signs of peritonitis.

Medical treatment aims to correct electrolyte and hematological imbalances while preventing the spread of infection. The choice of medications should be effective against both aerobic and anaerobic microorganisms and possess good intraperitoneal penetration. Antibiotic selection may be adjusted based on intraoperative bacteriological findings.

Laparoscopy is currently widely used as a preferred approach in the treatment of peritonitis [11]. It enables better exploration of the abdominal cavity, aspiration of peritoneal fluid, and treatment of the underlying cause with minimal abdominal wall trauma.

Ingestion of a single magnet does not typically cause specific injuries. However, when two magnets attract each other across a gastrointestinal wall, ischemia can occur, leading to perforation or fistula formation. In cases where two or more magnets have been ingested asynchronously, this results in the attraction of two digestive segments. When a single magnet is ingested asynchronously with a magnetic metal, the same mechanism apply, as magnetic attraction of the two foreign bodies will results in clinging of the intestinal wall and subsequent perforation. Endoscopic extraction may be impossible, necessitating surgical intervention. The therapeutic approach to acute peritonitis due to perforation depends on intraoperative findings. In our patient, a primary ileostomy was performed due to the duration of the clinical presentation and associated electrolyte and hematological disturbances. Other authors have performed intestinal resection followed by end-to-end anastomosis [4,7,8,12]. The post-treatment course is often favorable.

## 4 Limitations and strengths

One of the main limitations of our report was the failure to consider a foreign body as a potential cause of intestinal perforation in this patient initially. In emergency cases, the management approach depends on the patient's local and general conditions. However, despite this limitation, a two-step surgical intervention was performed in our patient, resulting in a favorable outcome.

## 5 Conclusion

Intestinal perforation caused by a digestive foreign body can occur in older children. The presence of a magnetic foreign body can be detected through imaging examinations and confirmed during emergency surgical exploration. In cases of delayed presentation, a two-step surgical approach may be considered.

## Consent for publication

The authors obtained written parental consent for publication.

## Ethical approval

Our case report received approval from the Ethics Committee.

## Funding

Our report did not receive any funding.

## Author contribution

Ndèye Aby NDOYE: Contributed to the conception, the design, and acquisition of data, drafted and revised the manuscript. She is the guarantor of the present work.

Ibrahima Bocar WELLE: Contributed to the acquisition of data. MMT: Contributed to the design and acquisition of data.

Bembo LAMEGA: Contributed to the conception, the design, and acquisition of data.

Amadou DIAWARA: Contributed to the conception, the design, and acquisition of data, drafted and revised the manuscript.

Florent Tshibwid A ZENG: Contributed to the conception, the design.

Gabriel Ngom: revised the manuscript.

## Guarantor

Prof. Ndèye Aby NDOYE.

## Research registration number

N/A

## Conflict of interest statement

The authors declare that they have no competing interests.

## Data Availability

The datasets used and analyzed during the current study are available from the corresponding author upon reasonable request.
